# The amount of hyaluronic acid and airway remodelling increase with the severity of inflammation in neutrophilic equine asthma

**DOI:** 10.1186/s12917-024-04136-2

**Published:** 2024-06-25

**Authors:** Nina Höglund, Heini Rossi, Hanna-Maaria Javela, Sanna Oikari, Petteri Nieminen, Anne-Mari Mustonen, Niina Airas, Vesa Kärjä, Anna Mykkänen

**Affiliations:** 1 Department of Equine and Small Animal Medicine, Faculty of Veterinary Medicine, University of Helsinki, Helsinki, FI-00014 Finland; 2 Department of Veterinary Biosciences, Faculty of Veterinary Medicine, University of Helsinki, Helsinki, FI-00014 Finland; 3 Institute of Biomedicine, School of Medicine, Faculty of Health Sciences, University of Eastern Finland, Kuopio, FI-70211 Finland; 4 Department of Environmental and Biological Sciences, Faculty of Science, Forestry and Technology, University of Eastern Finland, Joensuu, FI-80101 Finland; 5 Department of Clinical Pathology, Diagnostic Imaging Center, Kuopio University Hospital, Kuopio, FI-70210 Finland

**Keywords:** Asthma, Endobronchial biopsy, Fibrosis, Horse, Hyaluronan, Respiratory disease

## Abstract

**Background:**

Equine asthma (EA) is a chronic lower airway inflammation that leads to structural and functional changes. Hyaluronic acid (HA) has crucial functions in the extracellular matrix homeostasis and inflammatory mediator activity. HA concentration in the lungs increases in several human airway diseases. However, its associations with naturally occurring EA and airway remodelling have not been previously studied. Our aim was to investigate the association of equine neutrophilic airway inflammation (NAI) severity, airway remodelling, and HA concentration in horses with naturally occurring EA. We hypothesised that HA concentration and airway remodelling would increase with the severity of NAI. HA concentrations of bronchoalveolar lavage fluid supernatant (SUP) and plasma of 27 neutrophilic EA horses, and 28 control horses were measured. Additionally, remodelling and HA staining intensity were assessed from endobronchial biopsies from 10 moderate NAI horses, 5 severe NAI horses, and 15 control horses.

**Results:**

The HA concentration in SUP was higher in EA horses compared to controls (*p* = 0.007). Plasma HA concentrations were not different between the groups. In the endobronchial biopsies, moderate NAI horses showed epithelial hyperplasia and inflammatory cell infiltrate, while severe NAI horses also showed fibrosis and desquamation of the epithelium. The degree of remodelling was higher in severe NAI compared to moderate NAI (*p* = 0.048) and controls (*p* = 0.016). Intense HA staining was observed in bronchial cell membranes, basement membranes, and connective tissue without significant differences between the groups.

**Conclusion:**

The release of HA to the airway lumen increases in naturally occurring neutrophilic EA without clear changes in its tissue distribution, and significant airway remodelling only develops in severe NAI.

**Supplementary Information:**

The online version contains supplementary material available at 10.1186/s12917-024-04136-2.

## Background

Equine asthma (EA) is characterised by hyperresponsive airways and chronic lower airway inflammation of variable degree. Severe EA is associated with structural changes in the airways and apparent clinical signs, such as frequent coughing and respiratory effort at rest, while in mild/moderate EA the inflammation is milder without marked structural changes, and it is less distinct both in diagnostics and clinical signs. Typical clinical signs in mild/moderate EA are decreased performance and chronic, intermittent or occasional cough usually observed during exercise [1].

EA and human asthma share some etiological, pathophysiological, and immunological similarities, such as hyperresponsive airways, sensitivity to certain antigens and endotoxins, and pulmonary remodelling. Thus, horses can provide a naturally occurring translational model for human asthma [2]. While about 50% of severe human asthma is characterised by eosinophilia, this is unusual for severe EA, which typically shows neutrophilic response, and mild/moderate EA displays mildly increased neutrophils, eosinophils, or metachromatic cells [1]. Analysing histopathological findings in bronchial biopsies, horses with naturally occurring EA showed bronchial epithelial and submucosal inflammation, thickening of the basal membrane, airway smooth muscle hypertrophy, and fibrosis, expanding the understanding of the pathophysiology of EA [3–6].

Hyaluronic acid (HA) is a critical component of the healthy, effectively working extracellular matrix (ECM) with dual functions in inflammatory processes. HA is a non-sulfated glycosaminoglycan, produced in the inner leaflet of plasma membrane by the activity of HA synthases [7]. HA synthases and hyaluronidases are responsible for the diversity of HA molecular size. Different HA synthases produce HA molecules of various sizes, and hyaluronidases degrade HA to smaller fragments [8]. The biological activity of HA depends on its size: low-molecular-weight (LMW) HA (100–500 kDa) stimulates cell migration and inflammatory protein production, while high-molecular-weight (HMW) HA (1000–6000 kDa) has anti-inflammatory and tissue protecting functions [7, 9]. HA serves as a critical component in the ECM of most tissues, including airways and lungs [7, 10]. Elevated levels of HA in bronchoalveolar lavage fluid (BALF) have been detected in asthma in animal and human studies, and the presence of LMW HA has been suggested to indicate inflammation [11–15]. On the other hand, HA helps to bind water in tissues and to maintain elasticity, which contributes to the mechanical function of the airways. It also influences cell adhesion and proliferation and, therefore, HA potentially participates in airway remodelling [15–18]. While increased HA content has been reported in tracheal aspirates of horses with severe EA [19], the association of HA with naturally occurring EA and lung remodelling remains unclear.

This study investigated whether HA in equine airways and blood increases with neutrophilic airway inflammation (NAI) severity in horses with asthma, and explored the association of HA with airway remodelling. We hypothesised that the amount of HA in BALF, blood, and bronchi would differ between EA horses and control horses, and that the histological findings in bronchial biopsies would reflect the stage of NAI.

## Materials and methods

### Study population

This prospective clinical case-control study was conducted at the Department of Equine and Small Animal Medicine, Faculty of Veterinary Medicine, University of Helsinki. Sample size was calculated with the BALF neutrophil-% as a primary outcome, an estimated difference of 16 between the groups, an expected standard deviation (SD) of 20, a power of 80%, and significance at < 0.05. In total, 27 privately-owned adult asthma patients and 28 control horses and ponies were recruited. Control horses were living at the same farms as EA horses and were managed similarly. Inclusion criteria for EA horses included chronic (> 3 weeks) or recurrent cough. Inclusion criteria for the control horses were no history of chronic respiratory signs and no abnormal physical examination findings. Exclusion criteria for all horses included abnormalities in physical examination, airway endoscopy, airway cytology, blood haematology, or biochemistry indicative of other diseases than EA, such as infectious airway disease. Horses were divided into groups based on BALF neutrophil-%: ≤5% was considered as normal (controls), > 5–25% as moderate NAI, and > 25% as severe NAI [20].

### Sample collection

The examination and sample collection procedures included detailed history of the patient, physical examination, blood samples for haematology, fibrinogen concentration, biochemistry, and arterial blood gas analysis (collected anaerobically from the common carotid artery with the values normalised to rectal temperature), airway endoscopy, tracheal mucus score assessment, tracheal wash (TW), and BALF. Additionally, endobronchial biopsy (EBB) samples were collected from 30 patients.

After physical examination and blood analyses, horses were sedated with detomidine (0.01 mg/kg, Domosedan, Orion, Espoo, Finland) and butorphanol (0.005–0.01 mg/kg, Butordol, Intervet International, Boxmeer, The Netherlands), and the airways were locally anaesthetised with 40 mL of 1% lidocaine solution (Lidocain, Orion). A video endoscope (EC-3870LK, Pentax, Tokyo, Japan, length 170 cm, diameter 12.8 mm, instrument channel diameter 4.2 mm) was used for visualisation of the airways and mucus scoring (scale 0–5 [21], not performed blindly). TW was collected transendoscopically (20 mL) and BALF either transendoscopically from the right lung or by using a bronchoalveolar lavage (sterile 0.9% saline: 360 mL for horses and 240 mL for ponies) catheter (Equivet, Kruuse UK, Langeskov, Denmark). Two EBBs were obtained from the left bronchial tree with forceps (FB-245U, Olympus, Tokyo, Japan, EndoJaw Large oval shaped with needle, length 230 cm, diameter 2.45 mm) approximately 20–40 cm distally from main carina (from carinas 2.7–2.9 as described previously [22]). A single biopsy instrument was used for each animal.

### Airway cytology preparation and analysis

The TW and BALF samples (*n* = 55) were placed on ice and processed within one hour after collection. TW samples were centrifuged (300 × *g* for 10 min), and a smear was prepared from the cell pellet. The BALF samples for each horse were filtered through a single layer gauze, pooled, and cytocentrifuged (Thermo Scientific Cytospin 4 centrifuge, Thermo Scientific, Waltham, MA, USA) for cytology. TW and BALF slides were stained with May-Grünwald-Giemsa stain, and a blinded experienced investigator performed differential counts of inflammatory cells by counting 300 cells per slide for TW and 500 cells per slide for BALF. The supernatant (SUP) was extracted by centrifugation of BALF (first 300 × *g* for 10 min followed by the centrifugation of the supernatant at 3000 × *g* for 20 min), whereafter SUP was stored at − 80 °C.

#### HA-analyses

The lithium heparin plasma was separated by centrifugation (2450 × *g* for 10 min) and stored at − 80 °C. The plasma (*n* = 55) and SUP HA concentrations (*n* = 54) were measured with a sandwich-type enzyme-linked immunosorbent assay by a blinded person [23]. The detection limit of HA in SUP was 0.3 ng/mL, and samples with less HA were given the value zero. All samples were included in the data analyses. HA was also visualised, but not quantified, with confocal laser scanning microscopy (Zeiss Axio Observer equipped with the Zeiss LSM 800 confocal module, Carl Zeiss MicroImaging, Jena, Germany) in BALF and plasma. The subsamples were stained with CellMask Deep Red plasma membrane stain (Thermo Fisher Scientific) combined with Alexa Fluor 568-labeled HA binding complex (HABC) as previously outlined [23]. The molecular size distribution was assessed by a blinded person with Sephacryl S-1000 (1 × 30 cm) column with 100 mM ammonium bicarbonate as a buffer using previously pooled SUP samples from four horses in each diagnosis group [24]. The column was calibrated with 2500 kDa, 500 kDA, and 150 kDa HA standards (Hyalose, Oklahoma City, OK, USA). From each sample, 35 fractions (0.8 mL) were collected, and fractions 3–14 (HMW HA) and 15–35 (LMW HA) were combined and lyophilised. The dried samples were dissolved into 1% bovine serum albumin in phosphate buffered saline and analysed for their HA content.

#### Biopsy preparation and scoring

The EBBs (*n* = 30) were fixed in 10% neutral-buffered formalin for 48 h, whereafter processed routinely and embedded in paraffin wax. Sections were cut to 4 μm thickness and stained with haematoxylin and eosin (HE) and Masson Trichrome (Masson) stains for light microscopy. Light microscopy visualization for HA (*n* = 30) was also performed by utilising biotinylated HABC (bHABC) [25]. The specificity of the binding was insured with a negative control without the bHABC probe (Supplementary Material 1).

For the histological scoring, the HE- and Masson-stained sections were blindly assessed by an experienced veterinary pathologist (H-MJ). Prior to EBB scoring, the quality of each sample was classified to ascertain that it met the criteria for further scoring. The quality of all biopsy samples was evaluated using a quality scoring designed for the assessment of EBB smooth muscle mass by Bullone et al. (2014) [3]. Samples were considered to have sufficient quality when all required airway components were present (airway epithelium, ECM, and airway smooth muscle) with sufficient tissue architecture and orientation. After quality grading, the histological scoring system with 14 maximum points was used as described previously [4]. The assessment focused on changes in the endobronchial tissue potentially relevant for EA, such as epithelial hyperplasia and desquamation, goblet cell hyperplasia, thickening of the basal membrane, submucosal inflammation, presence of mucus glands within the lamina propria and among smooth muscle bundles, and airway smooth muscle fibrosis.

Another experienced pathologist (VK), unaware of the diagnoses, assessed the intensity of HA staining in respiratory epithelium, basal membrane, connective tissue, airway smooth muscle cell nucleus, cytoplasm, and cell membrane, endothelium of blood vessels, and mucus glands in the EBBs. The scoring used for the staining for HA was 0 = negative, 1 = faint, 2 = moderately positive, and 3 = strong positive staining for each location mentioned above. The scores were summed up for a total HA staining score (0–21).

### Statistical analyses

One-way analysis of variance was used for testing differences in age between the groups (control, moderate NAI, severe NAI). One-way analysis of covariance (ANCOVA) was performed for testing differences in continuous variables (partial pressure of oxygen in arterial blood (PaO_2_), blood fibrinogen concentration, TW neutrophil-%, BALF cytology, SUP HA, plasma HA, histological score, and biopsy HA staining score) between the groups. In the ANCOVA models, the age was added as a covariate to control the age difference between the groups. Normality was evaluated by graphical inspection of residuals from the model. Chi-square test was used for analysing differences between the groups in categorical variables (gender, breed, tracheal mucus score). Correlations between relevant variables (BALF neutrophil-%, TW neutrophil-%, tracheal mucus score, HA concentration in SUP, PaO_2_, histological score) were tested with Spearman’s rank correlation (*r*_*s*_) for all horses and separately for EA horses and control horses. Statistical analyses were performed using the IBM SPSS statistical package *v*27 (IBM, Armonk, NY, USA). The differences were considered statistically significant at *p* < 0.05. The results are presented as mean (± SD).

## Results

### Clinical variables, blood parameters, and airway cytology

General characteristics of the enrolled horses are presented in Table 1. The study population included 27 horses with NAI and 28 control horses. The most common clinical signs in horses with EA reported by owners were cough (*n* = 27), colorless to pale white nasal discharge (*n* = 20), and decreased physical performance (*n* = 16). The complete blood count or clinical chemistry values did not show any distinct deviations from reference ranges. From a total of 27 horses with EA, 10 (37%) had BALF neutrophil-% >25% and 17 (63%) had BALF neutrophil-% >5–25%. The mean retrieved BALF volume % from the total volume injected was 47 (± 12) % in control group, 46 (± 10) % in horses with moderate NAI, and 37 (± 10) % in horses with severe NAI. Horses with moderate NAI were older compared to control horses (*p* < 0.001), whereas there was no difference in age between control horses and horses with severe NAI (*p* = 0.109), or between horses with moderate NAI and horses with severe NAI (*p* = 0.149). Differences between control, moderate NAI, and/or severe NAI were found in PaO_2_, TW neutrophil-%, and BALF neutrophil-%, lymphocyte-%, macrophage-% and eosinophil-%, and tracheal mucus score after controlling for age (Table 1). In all horses, BALF neutrophil-% correlated positively with TW neutrophil-% (*r*_*s*_ = 0.779, *p* < 0.001), tracheal mucus score (*r*_*s*_ = 0.619, *p* < 0.001), and negatively with PaO_2_ (*r*_*s*_ = − 0.532, *p* < 0.001). In EA horses, BALF neutrophil-% correlated negatively with PaO_2_ (*r*_*s*_ = − 0.542, *p* = 0.004) and in control horses positively with TW neutrophil-% (*r*_*s*_ = 0.423, *p* = 0.025).

**Table 1:** Characteristics of control horses and horses with moderate neutrophilic airway inflammation (NAI) and severe NAI (mean ± SD)

	Control (*n* = 28)	Moderate NAI (*n* = 17)	Severe NAI (*n* = 10)	*P*
**Age ** *(years)*	11 ± 5^*a*^	16 ± 4^*b*^	13 ± 5^*ab*^	0.001
**Body weight ** *(kg)*	480 ± 123	453 ± 140	508 ± 144	0.593
**Sex ** *(n)*				0.138
Mare	10 (36%)	2 (12%)	2 (20%)	
Stallion	0	2 (12%)	0	
Gelding	18 (64%)	13 (76%)	8 (80%)	
**Breed ** *(n)*				0.960
Finnhorse	6 (21%)	3 (18%)	4 (40%)	
Standardbred	4 (15%)	2 (12%)	1 (10%)	
Warmblood	10 (36%)	6 (35%)	3 (30%)	
Icelandic	2 (7%)	2 (12%)	1 (10%)	
Pony	6 (21%)	4 (23%)	1 (10%)	
**Tracheal mucus ** *(0–5)*	1 ± 0.9^*a*^	3 ± 1.2^*b*^	3 ± 1.7^*b*^	< 0.001
**Blood fibrinogen ** *(g/L)*	3.3 ± 0.60	3.7 ± 0.64	3.9 ± 0.92	0.133
**PaO**_**2 **_ *(mmHg)*	102.3 ± 11.95^*b*^	93.2 ± 13.09^*b*^	79.1 ± 17.19^*a*^	< 0.001
**BALF**				
Total cells (/µl)	279 ± 157	312 ± 179	449 ± 311	0.094
Neutrophils (%)	2.1 ± 1.30^*a*^	13.9 ± 5.80^*b*^	44.2 ± 18.73^*c*^	< 0.001
Mast cells *(%)*	2.4 ± 1.98	312 ± 179	1.2 ± 0.87	0.098
Eosinophils *(%)*	0.2 ± 0.21^*a*^	1.6 ± 3.22^*b*^	0.3 ± 0.34^*ab*^	0.044
Lymphocytes *(%)*	50.2 ± 9.40^*b*^	52.4 ± 9.36^*b*^	34.6 ± 13.72^*a*^	< 0.001
Macrophages *(%)*	45.0 ± 9.68^*c*^	29.1 ± 7.19^*b*^	19.7 ± 8.85^*a*^	< 0.001

BALF = bronchoalveolar lavage fluid, PaO_2_ = partial pressure of oxygen in arterial blood. Dissimilar superscript letters indicate significant differences between the means within a row (ANCOVA, chi-square, *p* < 0.05)

### HA in SUP and plasma

**Fig. 1:**
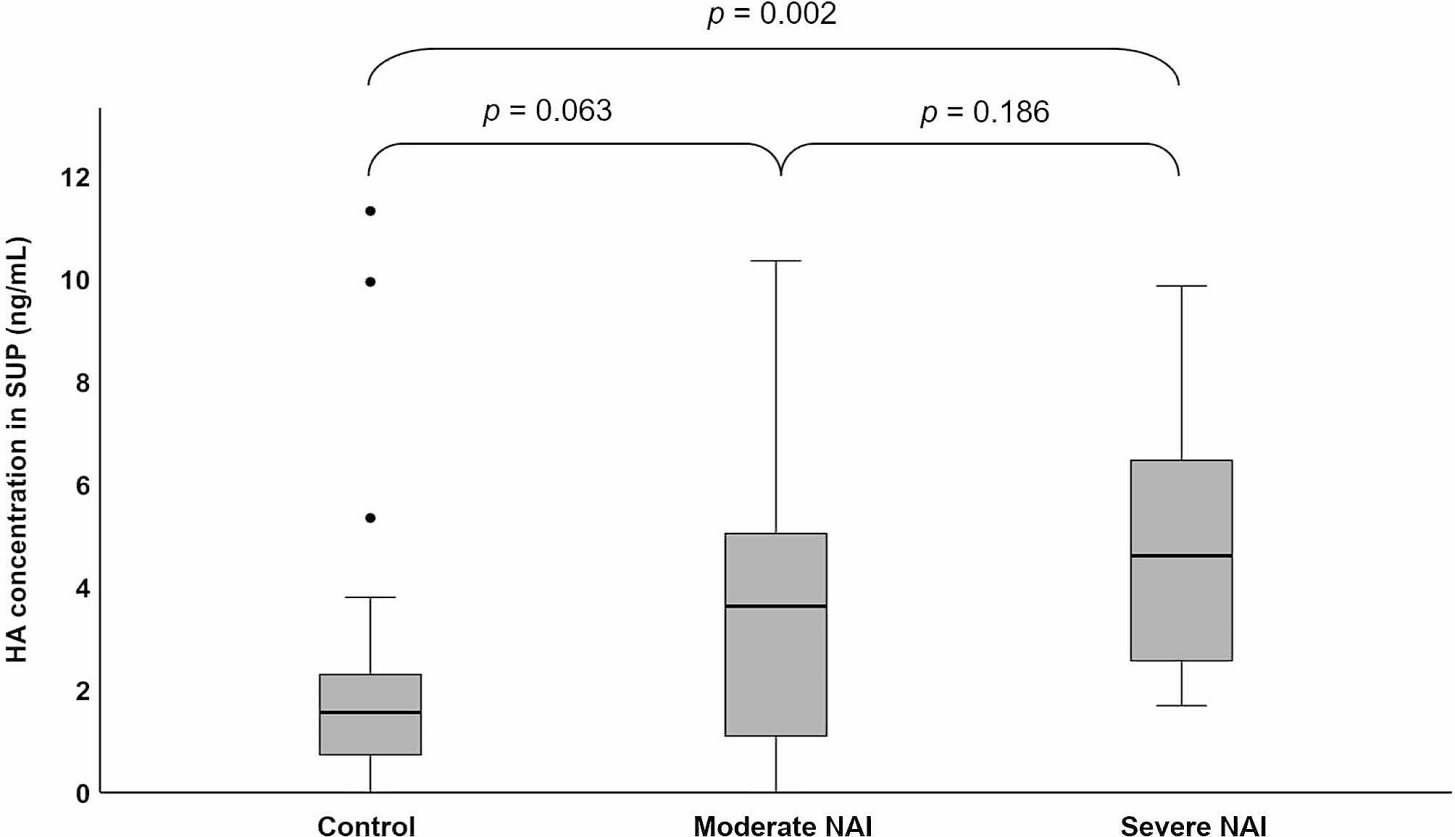
Boxplots of hyaluronic acid (HA) concentration in bronchoalveolar lavage fluid supernatant (SUP) in control horses (*n* = 28), horses with moderate neutrophilic airway inflammation (NAI, *n* = 16), and severe NAI (*n* = 10). Differences between groups are indicated (one-way ANCOVA)

**Fig. 2:**
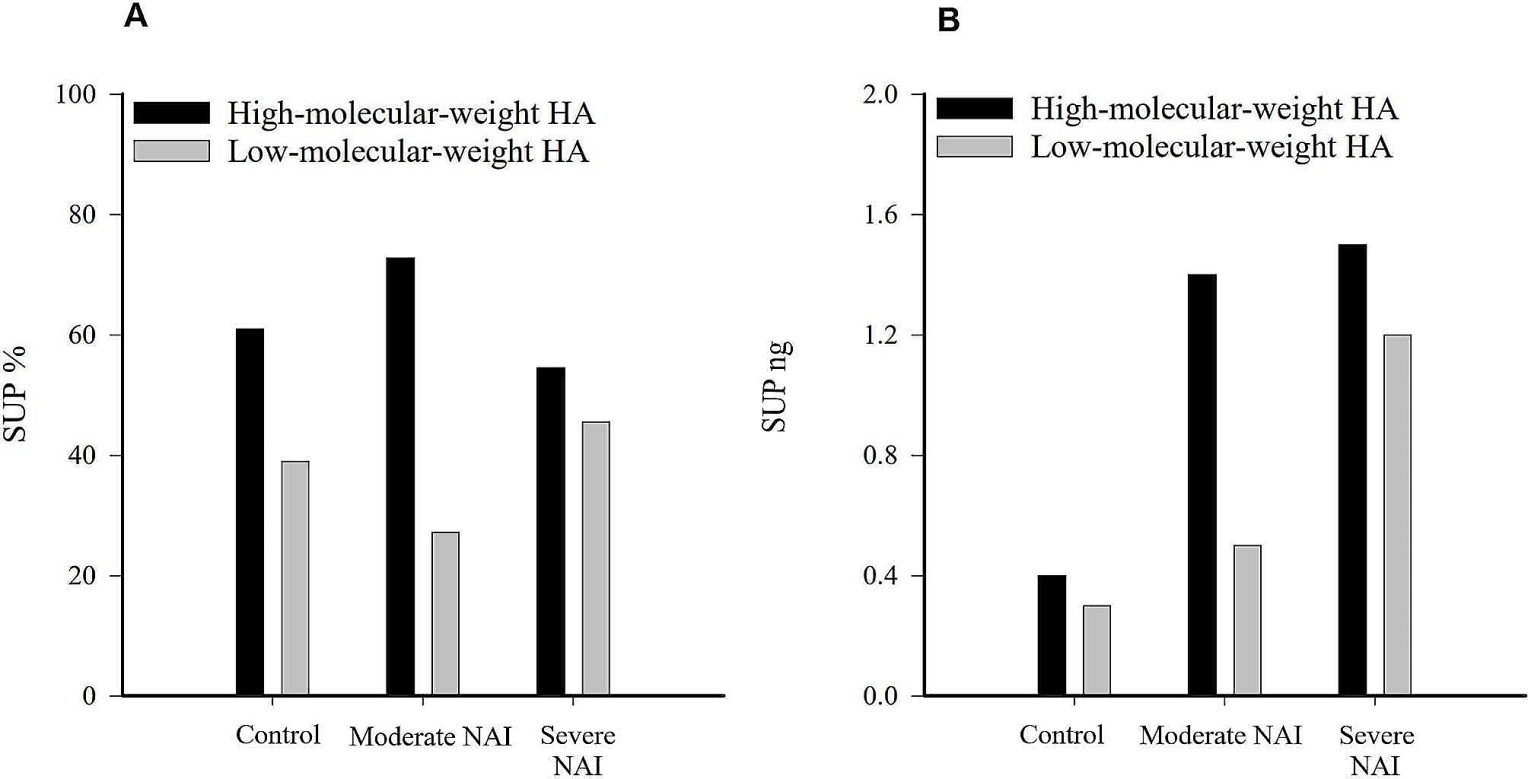
Visualisation of percentages (**A**) and amounts (**B**) of high-molecular-weight hyaluronic acid (HA) and low-molecular-weight HA in bronchoalveolar lavage fluid supernatant (SUP) in pooled control horses (*n* = 4), horses with moderate neutrophilic airway inflammation (NAI, *n* = 4), and severe NAI (*n* = 4)

SUP samples contained a relatively low level of HA, and the SUP HA concentration was below detection limit in some control horses (*n* = 4) and horses with moderate NAI (*n* = 3). The mean (± SD) HA concentration of SUP in control horses was 1.9 (± 2.00) ng/mL, in moderate NAI 3.4 (± 2.86) ng/mL, and in severe NAI 4.8 (± 2.59) ng/mL. The HA concentration in SUP was higher in EA horses compared to control horses (*p* = 0.007). In pairwise comparisons, HA concentrations in SUP were higher in horses with severe NAI compared to control horses (*p* = 0.002), while there was no difference between control horses and horses with moderate NAI (*p* = 0.063) or between horses with moderate NAI and severe NAI (*p* = 0.186) (Fig. 1). In all horses, HA concentrations in SUP correlated positively with BALF neutrophil-% (*r*_*s*_ = 0.459, *p* < 0.001), TW neutrophil-% (*r*_*s*_ = 0.474, *p* = 0.045), tracheal mucus score (*r*_*s*_ = 0.338, *p* = 0.012), and negatively with PaO_2_ (*r*_*s*_ = − 0.328, *p* = 0.016). Although the pooled data did not allow statistical testing, the amount and % of LMW HA tended to be higher in horses with severe NAI compared to controls and horses with moderate NAI when measured from a single pooled sample (Fig. 2). Similarly, the % of HMW HA tended to be higher in moderate NAI compared to controls and severe NAI.

**Fig. 3:**
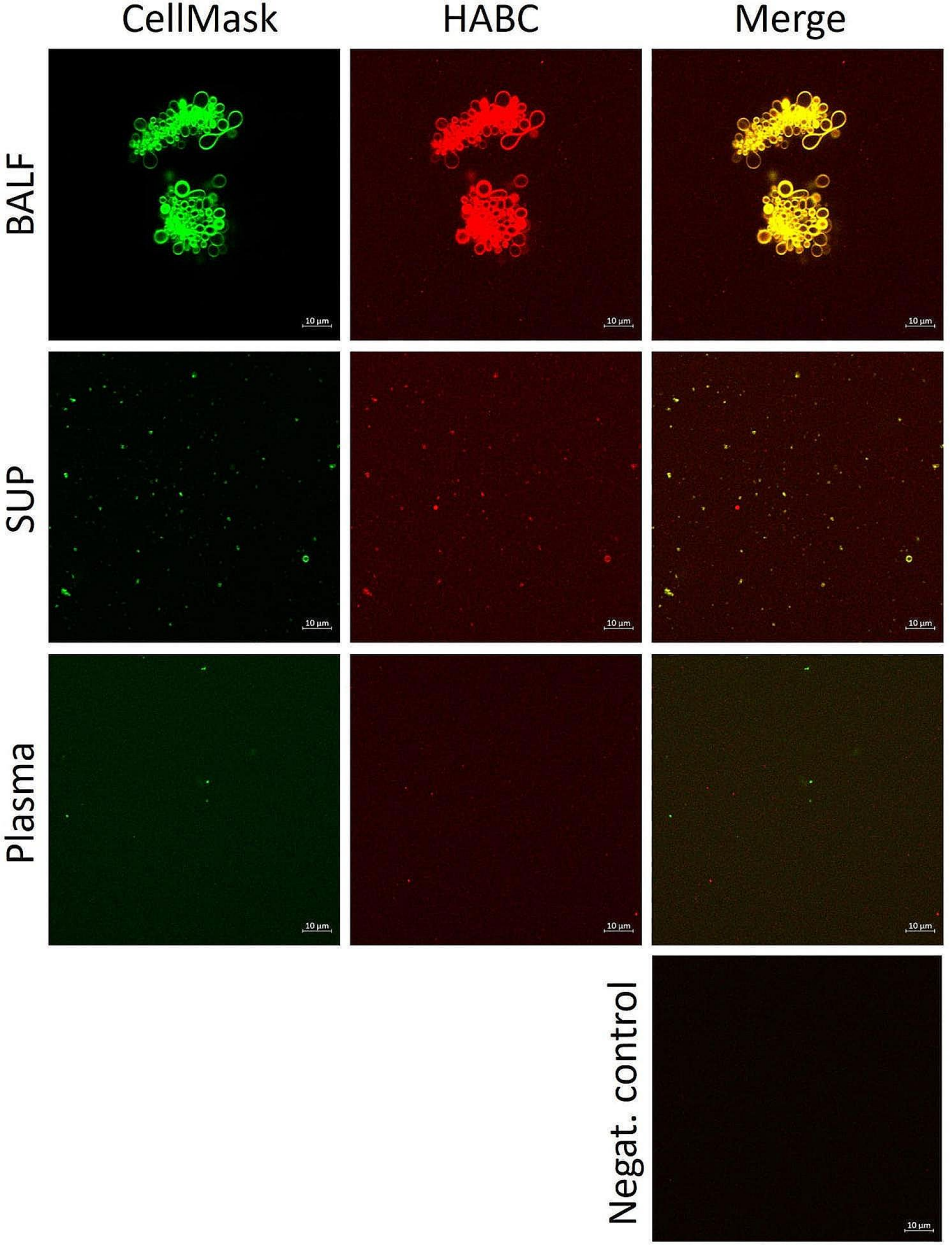
Visualisation of membrane structures and hyaluronic acid (HA) in bronchoalveolar lavage fluid (BALF), its supernatant (SUP), and plasma of horses with confocal laser scanning microscopy. The samples were stained with CellMask Deep Red plasma membrane stain (pseudocolored green) combined with Alexa Fluor 568-labeled HA binding complex (HABC, pseudocolored red). The BALF samples depict blebbing cells with both plasma membrane material and HA present

The mean HA concentration of plasma in control horses was 35.77 (± 78.99) ng/mL, in moderate NAI 22.65 (± 17.74) ng/mL, and in severe NAI 22.92 (± 26.44) ng/mL. The HA level in plasma did not differ between the groups (*p* = 0.869). With confocal microscopy, HA was visualised in BALF, SUP, and plasma samples. While HA was visualised bound to cell membranes, free HA was also present in all sample types (Fig. 3).

### Endobronchial biopsies and HA

**Fig. 4:**
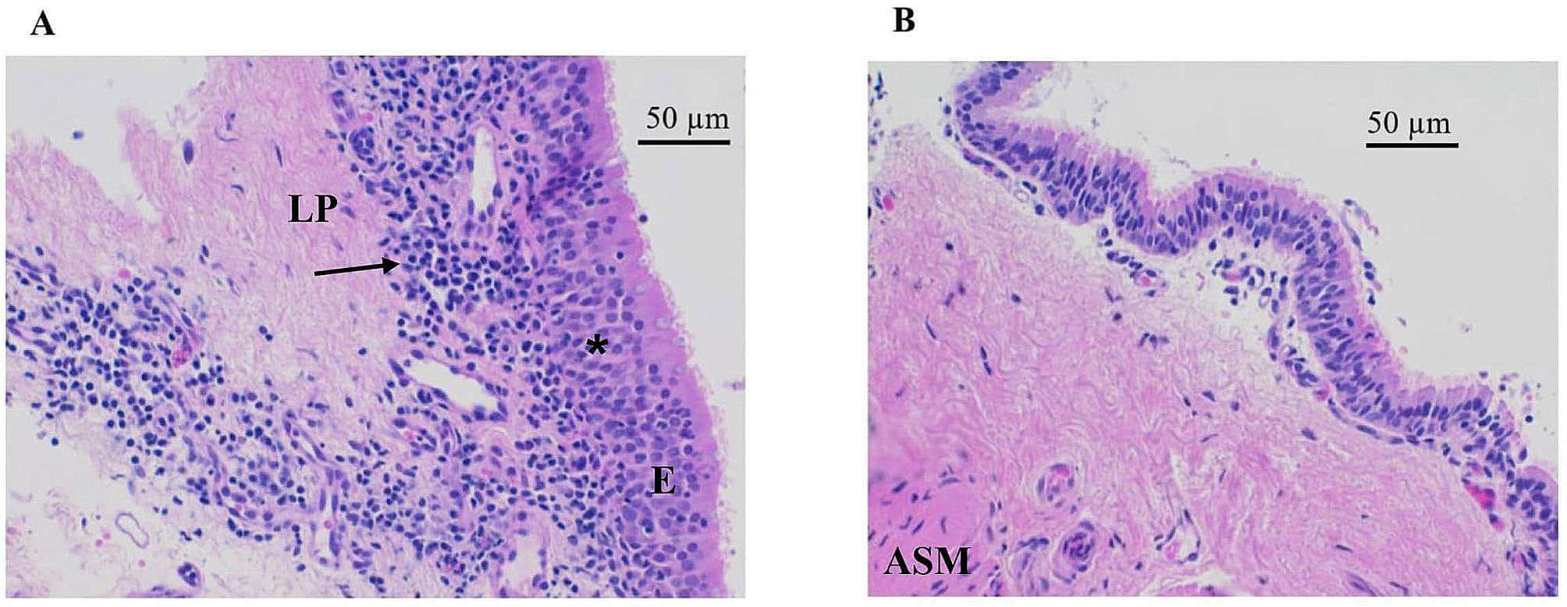
Endobronchial biopsy from a horse with severe neutrophilic airway inflammation (NAI) (**A**) and a control horse (**B**) stained with haematoxylin and eosin. There is marked epithelial hyperplasia (asterisk) and moderate inflammatory cell infiltrate (arrow) in superficial lamina propria (LP) in the horse with severe NAI (**A**) compared to the control horse (**B**). Airway smooth muscle (ASM) is not visible in panel A and only partially present in panel B. E = epithelium

**Fig. 5:**
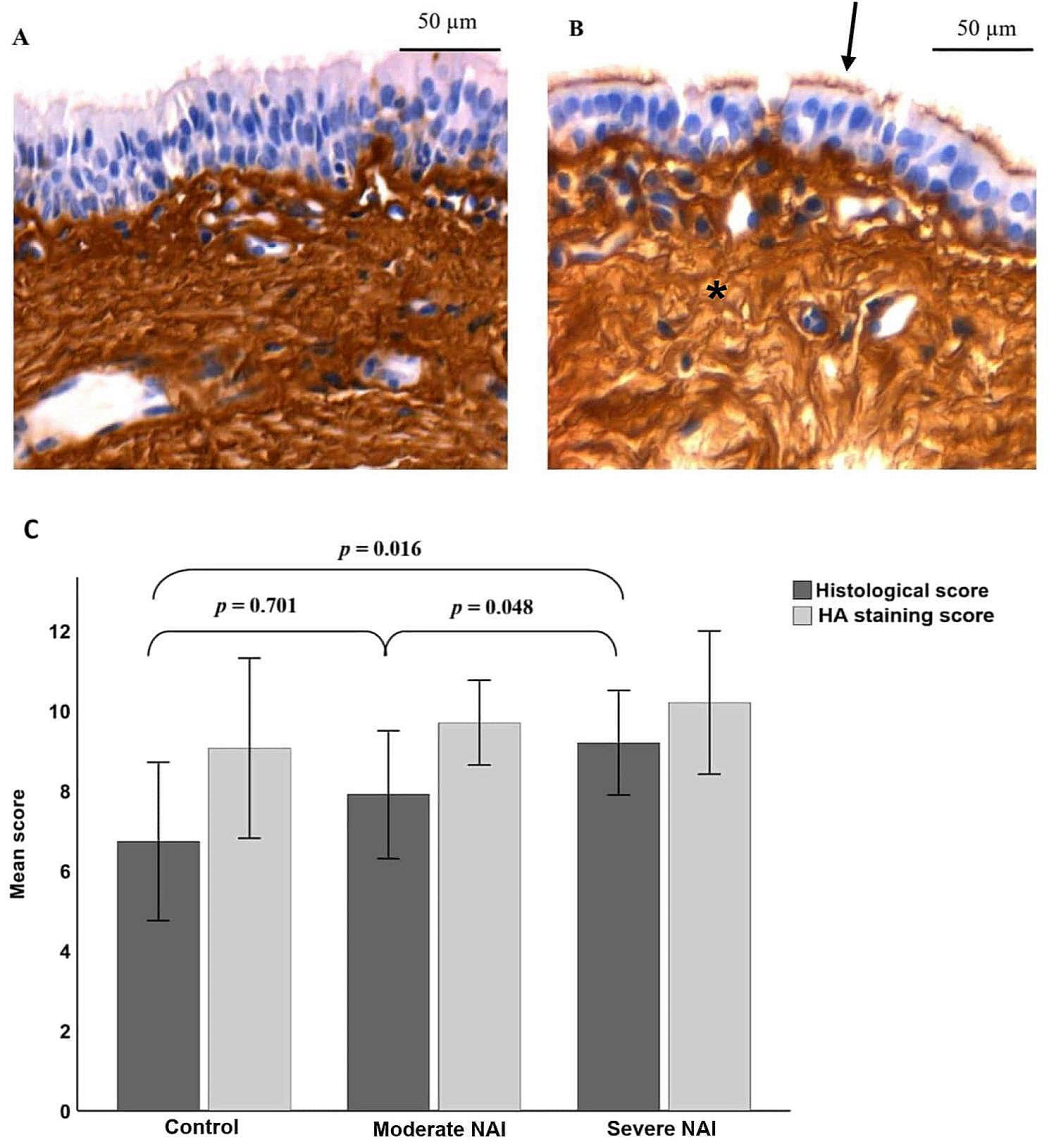
Endobronchial biopsies from a horse with severe neutrophilic airway inflammation (NAI) (**A**) and a control horse (**B**) stained with biotinylated hyaluronic acid (HA) binding complex. HA stained intensely in the epithelial cilia (arrow) and lamina propria (asterisk). The HA staining intensity was similar between control horses and horses with NAI (*p* = 0.561). Comparison of histological score and HA staining score in endobronchial biopsies in control horses (*n* = 15), horses with moderate NAI (*n* = 10), and severe NAI (*n* = 5) (means) (**C**). Error bars represent standard deviation (± SD). Differences in histology scores between groups are indicated (one-way ANCOVA)

All EBB samples were of sufficient quality, and the mean quality score was 2.5 (± 0.90) (1–5 [3]). In the EBBs, the most representative findings in EA horses were epithelial hyperplasia and moderate lymphoplasmacytic inflammatory cell infiltrate in epithelium and submucosa. Fibrosis of the airway smooth muscle and desquamation of the epithelium were only found in horses with severe clinical signs and abundant numbers of neutrophils in BALF (Fig. 4A). In control horses, the epithelium appeared thin and without any inflammatory cell infiltrate (Fig. 4B). The mean histological score was higher in severe NAI compared to moderate NAI (*p* = 0.048) and controls (*p* = 0.016) (Fig. 5C). One horse with severe NAI with a BALF neutrophil-% of 57.5% and TW neutrophil-% of 92.7%, and one horse with moderate NAI with a BALF neutrophil-% of 8.3% and TW neutrophil-% of 60.7% reached a histological score of 11, which was the highest score in our data. In all horses, the histological scores correlated positively with BALF neutrophil-% (*r*_s_ = 0.414, *p* = 0.023) and mucus in trachea (*r*_s_ = 0.382, *p* = 0.037). The negative correlation between the histological scores and PaO_2_ was of borderline significance (*r*_s_ = − 0.359, *p* = 0.051).

With the bHABC probe, a variable amount of HA was visualised in the cilia of endobronchial epithelium, cytoplasm of epithelial cells, and the basal membrane. HA was not present in the nucleus of respiratory epithelium, but the basal membrane and the underlying connective tissue stained intensely. No HA was visible in the cytoplasm or nuclei in the airway smooth muscle but, however, their cell membranes contained HA. The endothelium of blood vessels did not contain HA, and the mucus gland cytoplasm and membranes had only scant amounts of HA. There was no difference in HA staining intensity among groups (*p* = 0.561; Fig. 5).

## Discussion

This study investigated the potential significance of HA in the airways and remodelling of the bronchi in neutrophilic EA. The results suggest that the HA concentration in the epithelial lining fluid increases in EA, but the tissue distribution of HA remains the same regardless of the degree of inflammation. Furthermore, severe NAI results in distinct airway remodelling. Identifying associations between EA and HA content can offer new insights to EA pathophysiology and potentially also contribute to treatment in the future.

The increased HA in the SUP of horses with severe NAI suggests HA to have a role in neutrophilic airway inflammation in horses. Since the grouping in this study was not based on clinical signs, the relationship between the severity of clinical signs and HA in BALF could not be studied. The results agree with earlier studies with elevated HA in the BALF of humans with asthma, interstitial lung disease, acute respiratory distress syndrome, and smoke exposure [11, 13, 14, 26–29]. In these previous studies, the increased HA was suggested to derive from its increased production in lung interstitium fibroblasts, epithelial cells, and alveolar cells, and to be associated with tissue fibrosis. This could indicate an attempt of the organism to protect the tissues and to provide hydration during chronic inflammation. However, in this study, increased HA content was not associated with fibrosis. HA is synthesised in the inner surface of cell membrane by HA synthases, and after synthesis it is transported to the extracellular space [30]. Therefore, as shown in the present study, HA is abundant in cell membranes, extracellular structures, and connective tissue.

In this study, the tissue distribution of HA, the intensity of HA staining, and plasma HA concentration were similar between the groups, unlike in a study on human patients with induced asthma exacerbation [31]. Lauer et al. [31] found that controls had less HA in lung tissues due to lack of smooth muscle hypertrophy and basement membrane thickening. Additionally, in their study serum HA concentration peaked in asthmatic patients five hours after an allergen challenge, which was not performed in our study. Moreover, human allergic asthma is often eosinophilic, while we studied neutrophilic airway inflammation [32].

HA molecules can have both pro- and anti-inflammatory actions depending on their size, and during inflammation the production of LMW HA increases, which leads to the activation of inflammatory pathways and intensive cytokine production [7, 33]. It has been suggested that local production of LMW HA contributes to airway bronchoconstriction, inflammation, and fibrosis, and is associated with chronic asthma and remodelling [12, 17, 18]. This is supported by our results, where LMW HA tended to increase in SUP samples of horses with severe NAI. HA consists of repeated disaccharide units in a coiled chain that provides excellent viscoelasticity and a significant ability to retain water [7]. The HMW HA can potentially protect the intercellular collagen and elastin from degradation, enhance cell migration and proliferation in the regeneration process, and promote elimination of useless or harmful components [7, 34]. Theoretically, HMW HA could also promote the remodelling process by stimulating cell proliferation and migration. Nevertheless, the role of HMW HA in airway remodelling remains unclear. The tentative increase of HMW HA, especially in horses with moderate NAI, could be considered a protective mechanism in moderate airway inflammation [15, 35, 36], while the higher levels of LMW HA in severe NAI suggest intensified production and degradation of HA to smaller fragments during progressive inflammation [18]. Future studies could focus on investigating the presence and roles of LMW HA, HMW HA, and related hyaluronidases in EA. Results on the production and degradation of HA during NAI could offer interesting insights regarding EA therapy.

In a study by Esposito et al. [37], patients with acute lung injury and hypoxemia had increased HA content in BALF. Similarly, in our study, increased airway neutrophilia and HA concentration were associated with lower arterial oxygen content indicating impaired lung function. Ernst et al. [29] detected increased BALF HA levels in patients with interstitial lung disease, and an inverse relationship between HA levels and diffusion capacity indicating impaired gas exchange. Likewise, our results showed a relationship between elevated HA in SUP and impaired oxygen transfer from alveoli to blood.

The most pronounced changes in EBBs in EA horses were observed in the bronchial epithelium and superficial submucosa. Bullone et al. [4] reported the epithelial and submucosal inflammatory infiltrates to be the most important factors differentiating EA horses in exacerbation from those in remission. This suggests that active inflammation occurs in superficial bronchial tissues. This is also thought to be a site where inflammation is initiated. However, in human asthma, airway smooth muscle has also been suggested to participate in the initiation of inflammation [38]. The significantly higher histological scores in severe NAI compared to controls are in line with the results of Bullone et al. [4]. However, the mean histological score for all groups was higher in our study compared to the Bullone et al. [4] study, where a cut-off score for EA horses was more than five. Bullone et al. [4] used hyoscine butylbromide to relax the airway smooth muscle prior to EBB sampling, which might have affected the tissue architecture of EBB samples.

Our results suggest positive associations between histological scores, airway neutrophilia, and the amount of mucus in the airways. Recent studies have also reported conflicting results on the reliability of EBBs in distinguishing horses with severe EA from control horses, however, the sampling site in these studies was more proximal, closer to the main carina [39, 40]. The nonsignificant differences in the histological scores between horses with moderate NAI and control horses in the present study may be related to the low level of neutrophilia in horses with moderate NAI. However, Bessonnat et al. [6] reported conflicting results, where tissue remodelling was significant also in horses with mild/moderate EA. In our data, the horses with moderate NAI had lower neutrophil-% compared to the study by Bessonnat et al. [6], which could have affected the severity of remodelling. Dupuis-Dowd & Lavoie [5] showed results supporting bronchial smooth muscle remodelling also in mild/moderate forms of the condition. Their study focused on the airway smooth muscle characteristics and reported that horses with mild/moderate EA did not have smooth muscle hyperplasia or hypertrophy, but the expression of a hypercontractile smooth muscle myosin chain isoform was greater in horses with mild/moderate EA compared to control horses.

There are some limitations to this study. The grouping of EA horses was based on airway cytology (not EA phenotype), and only NAI was considered, while some horses had concurrent eosinophilic or metachromatic cell airway inflammation. Environmental factors, such as stabling conditions and transport prior to examinations and sampling, could not be controlled due to the nature of the study with client-owned horses. This might have influenced the clinical status and airway neutrophilia. In addition, some EA horses in remission could have been grouped incorrectly as controls. The small number of animals in the EBB evaluation could have influenced the results of histological and HA scores. Due to technical reasons, the HA molecular size could be measured only from pooled samples, which did not allow statistical testing. A correction for the dilution in BALF was not performed with a dilution marker, since it did not affect the BALF results in our previous study [41].

## Conclusion

In this study, we show for the first time that HA concentration increases in BALF in naturally occurring neutrophilic EA. However, the HA tissue distribution and plasma concentration remained similar in control horses and horses with EA. Airway remodelling was the highest in horses with severe NAI, but it was not associated with airway HA content. Identifying the association between EA and increased HA concentration in lungs can offer new insights to EA therapy modalities and further research. In the future, the roles of HMW and LMW HA in EA need further investigations with the focus on therapy.

### Electronic supplementary material

Below is the link to the electronic supplementary material.

Supplementary Material 1: Endobronchial biopsy from a control horse stained with (A) or without (B) the biotinylated hyaluronic acid binding complex

## Data Availability

The datasets used and/or analysed during the current study are available from the corresponding author on reasonable request.

## Abbreviations

ANCOVA: analysis of covariance
BALF: bronchoalveolar lavage fluid
bHABC: biotinylated hyaluronic acid binding complex
EA: equine asthma
EBB: endobronchial biopsy
ECM: extracellular matrix
HA: hyaluronic acid
HABC: hyaluronic acid binding complex
HE: haematoxylin and eosin
HMW: high-molecular-weight
LMW: low-molecular-weight
Masson: Masson Trichrome
NAI: neutrophilic airway inflammation
PaO_2_: arterial oxygen content
r_s_: Spearman’s rank correlation
SD: standard deviation
SUP: supernatant
TW: tracheal wash
