# Supplementary figures and images for: The amount of hyaluronic acid and airway remodelling increase with the severity of inflammation in neutrophilic equine asthma
Source: BMC Vet Res. 2024 Jun 25;20:273. doi: 10.1186/s12917-024-04136-2 (PMC11197223; doi:10.1186/s12917-024-04136-2)

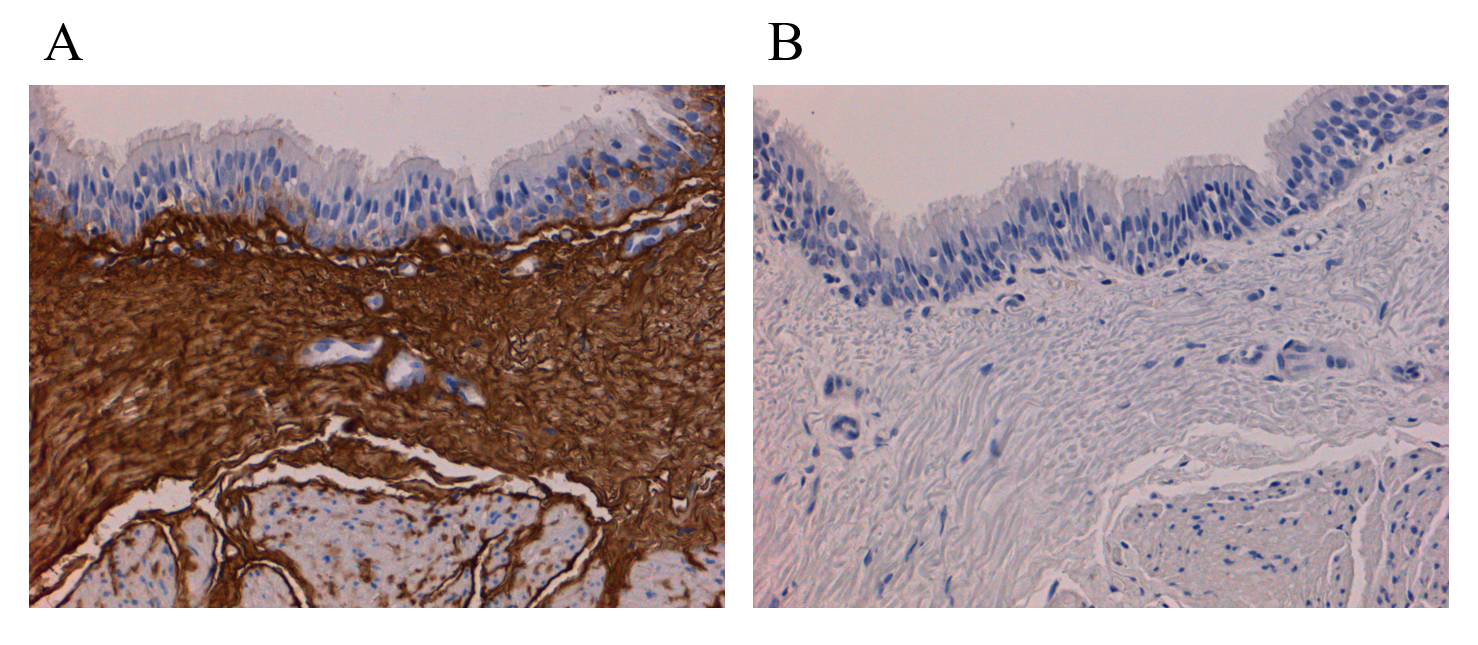

Supplement: Supplementary file 1 — Supplementary Material 1: Endobronchial biopsy from a control horse stained with (A) or without (B) the biotinylated hyaluronic acid binding complex [file 12917_2024_4136_MOESM1_ESM.png]
